# The Michigan Hand Outcomes Questionnaire (MHQ-Swe) in patients with distal radius fractures—cross-cultural adaptation to Swedish, validation and reliability

**DOI:** 10.1186/s13018-021-02571-7

**Published:** 2021-07-07

**Authors:** J. Blomstrand, J. Karlsson, M. Fagevik Olsén, G. Kjellby Wendt

**Affiliations:** 1 Department of Occupational Therapy and Physiotherapy, Sahlgrenska University Hospital, Göteborgsvägen 31, Mölndal, SE-431 80 Sweden; 2 Institute of Clinical Sciences, Department of Orthopaedics, Sahlgrenska Academy, University of Gothenburg, Gothenburg, Sweden; 3 Department of Orthopaedics, Sahlgrenska University Hospital, Göteborgsvägen 31, Mölndal, SE-431 80 Sweden; 4 Institute of Neuroscience and Physiology, Department of Health and Rehabilitation - Physiotherapy, Sahlgrenska Academy, University of Gothenburg, Gothenburg, Sweden

**Keywords:** Cross-cultural adaptation, Swedish translation, Michigan Hand Outcomes Questionnaire, Distal radius fracture, Hand therapy, Occupational therapy, Rehabilitation, Activity performance

## Abstract

**Background:**

The Michigan Hand Outcomes Questionnaire—MHQ—is a well-known self-assessment questionnaire, where patients’ own perception in terms of recovery, pain and the ability to return to activities of daily living is assessed. The purpose of the study was to translate and culturally adapt the Michigan Hand Outcomes Questionnaire to Swedish and to test the validity and reliability in patients with surgically treated distal radius fractures.

**Methods:**

The cross-cultural adaptation and the translation process were conducted according to predefined guidelines. Seventy-eight patients with surgically treated distal radius fractures completed the translated version of the questionnaire on their six-week follow-up visit.

**Results:**

The translation and cross-cultural adaptation process revealed no major linguistic or cultural issues. The internal consistency of the MHQ-Swe ranged from 0.77 to 0.94 at test 1 and from 0.81 to 0.96 at test 2 for all subscales, which indicates good internal consistency in the subscales. The hand function subscale revealed the lowest results and work performance the highest. The ICCs showed excellent test-retest reliability, ranging from 0.77 to 0.90 on all MHQ subscales and 0.92 on total score. The highest results for the ICC were seen in the satisfaction subscale (ICC = 0.90), while the lowest were seen in the aesthetic subscale (ICC = 0.77). The correlation analysis between the MHQ-Swe, PRWE and VAS showed a generally moderate to high correlation for all the subscales.

**Conclusions:**

The Swedish version of the MHQ, the MHQ-Swe, showed good validity and reliability and it is therefore an appropriate and relevant questionnaire for use in patients with surgically treated distal radius fractures.

**Trial registration:**

FoU i VGR, Projectnumber: 208491, registered December 9, 2015.

## Introduction

A distal radius fracture (DRF) is one of the most common skeletal injuries [1]. The incidence rate in Sweden has been reported as 31–32 per 10,000 person-years [2, 3]. Some of the fractures are considered unstable and require surgical stabilisation [4]; fixation of the fracture using a volar plate is a common method [5]. The proportion of DRFs treated surgically in Sweden is between 20 and 30% [2]. After surgery, postoperative rehabilitation is an essential part of the treatment of a surgically treated DRF, due to the risk of long-term disability [6]. As the hands are extremely important in several aspects of human life, individuals with hand injuries can experience both physical, psychological and social consequences [7]. The outcomes after a hand injury are evaluated in different ways, using both objective and subjective measurements. Even though there is no consensus in terms of outcome assessment, pain, grip strength, range of motion (ROM), reported complications and radiographs are considered to be the most important outcome measurements [8]. In terms of hand rehabilitation, the identification of functional goals that are meaningful for the patient is important, along with the coaching of patients to enable them to be independent in self-care as soon as possible [7]. It is crucial to evaluate outcomes in a patient-centred rehabilitation and patient-reported outcome measurements (PROMs) are a valuable and important aspect.

The use of PROMs after a DRF provides important information in terms of the patient’s subjective perception of hand function, activity level and final outcome after surgery. When compared with traditional measurements evaluating outcomes, such as ROM or the presence of complications, PROMs add another dimension to the outcomes and the treatment efficiency and patient well-being [8]. PROMs can be used to support the clinical management of patients, assess provider performance and provide a basis for evaluative research and they are an important factor when it comes to increasing patients’ participation in their care and incorporating patients’ experiences in improving rehabilitation models and evaluation processes.

The Michigan Hand Outcomes Questionnaire, the MHQ [9], is regarded as one of the most common PROMs to assess patients’ own perceptions in terms of recovery, pain and the ability to return to activities of daily living [8]. It is used to assess the patient’s estimation/perception of his/her hand function, pain, satisfaction with hand function and aesthetics of the hand [9]. The MHQ has several unique characteristics. First, it assesses each hand separately. Second, it addresses questions about aesthetics and, third, it also assesses the patients’ satisfaction with overall function, motion and strength, for example, which is not covered by other questionnaires. The MHQ has shown good reliability, validity and responsiveness for clinical change in patients with a DRF and other hand injuries and it has been translated and culturally adapted to several different languages, including Farsi, German, Japanese, Korean, Portuguese and Turkish [9–17]. There is, however, no existing translation into Swedish.

When translating a questionnaire, it is important not only to translate it but also to adapt the questionnaire cross-culturally and evaluate its validity and reliability in different patient groups. Activities of daily living (ADL) are closely related to the use and function of the hand and wrist. Sociocultural and linguistic differences in the activities of daily living could affect the validity of the translated questionnaire and it is crucial to have a linguistically and culturally well-adapted process [13]. The purpose of the study was to translate and culturally adapt the MHQ to Swedish and to test the validity and reliability in patients with surgically treated distal radius fractures.

## Material and methods

Permission to use and translate the MHQ [9] was obtained from the developers (academic licence #3372). The regional ethical review board in Gothenburg, Sweden, approved the study (registration number #1157-16). The study was conducted at an orthopaedic hand therapy unit at a university hospital in the southwestern part of Sweden, between January 2016 and April 2019. The study was divided into two parts. First, the translation and the cross-cultural adaptation were performed. Second, the reliability and validity of the Swedish version of the MHQ was tested.

### Instruments

The Michigan Hand Outcomes Questionnaire—MHQ—was developed in 1998 by Chung et al., with the goal of creating a questionnaire which could be used to measure outcomes for patients with hand injuries [9]. The MHQ is a self-reported questionnaire consisting of 37 questions in six domains: (I) overall hand function (five questions per hand), (II) activities of daily living (ADL) (five questions per hand and seven questions on two-handed activities), (III) work performance (five questions), (IV) pain (five questions per hand), (V) aesthetics/appearance (four questions per hand) and (VI) patient satisfaction with hand function (six questions per hand). Apart from work performance, all the subscales are administered separately for the right and left hand. The item responses range from 1 to 5.

The raw score for each subscale is the sum of the responses for each scale item, which is converted to a score ranging from 0 to 100. The total scores in the subscales range from 0 to 100, with a higher score indicating a better result, apart from pain. On the pain scale, high scores indicate more pain. Depending on which hand is affected, the right- or left-hand score on each subscale is selected to calculate the total score on the subscale. If both hands are affected (for instance, in rheumatoid arthritis), the right- and left-hand scores are averaged. A total score is calculated by adding up the total scores on the subscales after reversing the pain scale score and then dividing by 6 [9].

The Patient Rated Wrist Evaluation (PRWE) was developed in 1996 by MacDermid [18, 19]. The PRWE is a self-reported questionnaire incorporating areas of pain and function, where the function is divided into specific activities and general activities. The pain subscale consists of five questions about when and how often the patient experiences pain, while the subscale for function consists of 10 questions about activities (six questions about specific activities, and four questions about general activities) about how to manage various everyday activities. Each question is answered on a 11-level scale, from 0 (no pain/no symptoms) to 10 (worst pain imaginable/impossible to do), where patients assess their status. The scores for the five pain-related questions are added up to produce a maximum of 50 points. The scores from the 10 questions related to function are also summarised but divided by two, also adding up to a maximum of 50 points. In total, the range of the score is 0 to 100, where 0 is interpreted as no discomfort/pain, and 100 as maximum discomfort/pain. A loss of response is replaced by the average of the patients’ other answers on the current subscale [20]. The PRWE has been tested for reliability and validity in Swedish settings [20] and is regarded as one of the most useful questionnaires for assessing outcome following a DRF [21, 22].

Pain was measured on a VAS [23], with two questions related to the experience of pain ranging from 0 (no pain) to 10 (worst imaginable pain). The patients estimated their pain numerically.

### Translation and cross-cultural adaptation

The translation and cross-cultural adaptation were based on pre-defined guidelines formulated by Beaton et al. [24]. Two professional, native Swedish-speaking translators independently translated the questionnaire from English into Swedish. The two translators, together with two of the authors, then synthesised the results of the two translations. This version was then independently translated back to English by two other, native English-speaking translators. The two back-translators had not read the original MHQ in English and were not aware of the purpose of the study. The back translations were reviewed by two of the authors to ensure intelligibility and consistency with the original version. A committee of experts then met to review all the versions (the original version in English, the synthesis of the translations into Swedish and the back-translations into English) to develop a preliminary version of the questionnaire.

The preliminary version of the questionnaire, the MHQ-Swe, was tested in a clinical setting in 40 patients. The final version was completed and every part of the translation and cross-cultural adaptation process was presented to the developers of the questionnaire. The final version of the questionnaire was then tested for psychometric properties.

### Reliability and validity-testing

The patients were recruited consecutively at an orthopaedic hand therapy unit. Patients, aged 18 years and over, with a distal radius fracture, all treated surgically using a volar plate, who spoke and understood Swedish in speech and writing, were asked to participate in the study in connection with a return visit to an occupational therapist (OT) 6 weeks post-surgery. All the participants gave their written, informed consent to participate in the study. Patients with other injuries/diseases in the hand/arm/shoulder that could have affected their activity performance or had diagnosed dementia or some other cognitive impairment were excluded.

At the visit, the OT collected demographic and clinical data, which included aspects of age, gender, hand dominance, information regarding the injury and the VAS, MHQ-Swe and PRWE.

Seventy-eight patients participated in phase 2. The demographics of the 78 participants in phase 2 are presented in Table 1.

**Table 1:** Characteristics of the participants (n = 78), mean (±SD) or number of patients (%)

	n		
Age, years	61.1 (13.5)		
Gender, female	63 (81%)		
	Right	Left	Both
Hand dominance	69 (89%)	8 (10%)	1 (1%)
Injured hand	35 (45%)	42 (54%)	1 (1%)
	Yes	No	Both
Injury to dominant hand	40 (51%)	36 (46%)	2 (3%)

Reliability is a consistency measurement and can be described in terms of reproducibility and internal consistency. The internal consistency assesses the consistency of the respondent’s answers across items within a questionnaire and is important when it comes to evaluating the correlation between the questions on each subscale.

A test of reproducibility was performed through a test-retest, where patients filled in the MHQ-Swe twice, once at their 6-week postoperative visit, and once at home 5 to 7 days after the visit. The last questionnaire was then returned by mail. At the 6-week postoperative visit, the patients also completed the PRWE and estimated their pain on a VAS in order to test the validity of the questionnaire. The construct validity was assessed by comparing the subscales of the MHQ-Swe with the Swedish version of the Patient Rated Wrist Evaluation (PRWE) [20] and the VAS [23].

### Statistics

The collected data were analysed with SPSS software (version 27).

Descriptive statistics were used to describe the participants’ characteristics.

The calculations in the current study were, where applicable (subscales I, II, IV, V and VI), performed on the injured hand and not on the right and left hand.

Internal consistency was assessed using Cronbach’s alpha. A higher coefficient (range 0–1) indicates a more consistent scale. A threshold value of 0.80 was considered acceptable.

Test-retest was assessed using an intraclass correlation coefficient (ICC). The classification suggested by Cicchetti et al. [25] as poor (< 0.40), fair (0.40–0.59), good (0.60–0.74) and excellent (0.75–1.00) was used [25].

The association between the domains in the instruments was calculated using Spearman’s rank correlation. In interpreting the correlation strength, the limits of r = 0 for no correlation, r = 0.1–0.3 for weak correlation, r = 0.4–0.6 for moderate correlation, r = 0.7–0.9 for strong correlation and r = 1 for perfect correlation were used [26].

## Results

### Translation and cross-cultural adaptation

The translation and cross-cultural adaptation process revealed no major linguistic or cultural issues. Some minor discrepancies regarding cultural differences were encountered. For example, the question relating to “Turn a door knob” was altered to the Swedish wording for “att skruva i en glödlampa” (screw in a light bulb) after the translation process and expert group, as door handles are used instead of knobs in Sweden. Moreover, the question about “Carry a grocery bag” was altered, because this activity is not two handed in Sweden.

Forty patients (8 men, 32 women, mean age 52.6) with surgically treated distal radius fractures participated in phase one, testing of the pre-final version of the questionnaire, and they were asked whether any question appeared to be unclear, if something was missing or needed to be added. Most of the patients had no comments or questions about the questionnaire, but some of the patients addressed some issues. Mainly, some of the older women argued that it was difficult to answer the question about “your normal work” due to retirement. This question was altered to suit patients who were retired as well.

### Validity and reliability testing

The average scores for the instruments are presented in Table 2. Regarding the MHQ, the lowest scores (indicating more disability) were seen on the work performance subscale (36.7 in T1, 44.0 in T2) and the highest (indicating lesser disability) on the aesthetics subscale (77.5 in T1, 76.0 in T2).

**Table 2:** Average scores as the mean (SD)

	T1 n = 78	T2 n = 78
**MHQ (injured hand)**		
I. Overall hand function	53.8 (16.4)	56.4 (15.5)
II. ADL	62.3 (21.6)	68.2 (21.0)
III. Work performance	36.7 (23.6)	44.0 (25.3)
IV. Pain	64.2 (20.5)	64.4 (21.2)
V. Aesthetics^a^	77.5 (22.1)	76.0 (24.2)
VI. Satisfaction	58.2 (21.6)	59.2 (20.6)
Total	58.6 (16.5)	61.0 (17.1)
**PRWE**		
Pain	18.6 (9.8)	
Activity	21.3 (11.3)	
Total	39.8 (19.5)	
**VAS**	25.5 (20.4)	

T1, test; T2, retest; ADL, activities of daily living

^a^T1 n = 77, T2 n = 76

The internal consistency of the MHQ-Swe, estimated by the internal consistency coefficient Cronbach’s alpha, ranged from 0.77 to 0.94 on test 1 (T1) and from 0.81 to 0.96 on test 2 (T2) for all subscales, which indicates a good internal consistency on the subscales (Table 3). The hand function subscale revealed the lowest results (0.77 on T1 and 0.81 on T2), while work performance revealed the highest results (0.94 on T1 and 0.96 on T2).

**Table 3:** Internal consistency and test-retest reliability of the Swedish version of the MHQ n = 78

MHQ subscale	Number of items	Cronbach’s alpha		Intraclass correlation coefficient		
		T1	T2	ICC	95% CI	p value
I. Hand function	5	0.77	0.81	0.83	0.73–0.89	< 0.001
II. ADL	12	0.93	0.94	0.87	0.78–0.93	< 0.001
III. Work performance	5	0.94	0.96	0.84	0.72–0.91	< 0.001
IV. Pain^a^	5	0.85	0.87	0.86	0.78–0.91	< 0.001
V. Aesthetics^b^	4	0.79	0.82	0.77	0.63–0.85	< 0.001
IV. Satisfaction	6	0.89	0.87	0.90	0.84–0.94	< 0.001
Total				0.92	0.87–0.95	< 0.001

T1, test; T2, retest; SD, standard deviation; ICC, intraclass correlation coefficient; CI, confidence interval

^a^T1 n = 72, T2 n = 73

^b^T1 n = 77, T2 n = 76, ICC n = 76

The ICCs showed excellent test-retest reliability, ranging from 0.77 to 0.90 on all MHQ subscales, and 0.92 on total score. The results of the intraclass correlation for the test-retest are shown in Table 3. The highest results for the ICC were found for the satisfaction subscale (ICC = 0.90) and the lowest for the aesthetic subscale (ICC = 0.77).

The correlation analysis between the MHQ-Swe, PRWE and VAS generally revealed a moderate to high correlation for all the subscales (Table 4). The MHQ subscale for pain had a strong correlation with the VAS and the PRWE pain subscale and the correlation was also strong between the MHQ subscale of ADL and the activity subscale and total score on the PRWE and between the total scores on the MHQ and PRWE. Weak correlations were found between the MHQ subscale for aesthetics and the VAS, while moderate correlations were found between the MHQ subscale for aesthetics and all the subscales in the PRWE.

**Table 4:** The construct validity measured with Spearman’s correlations for the MHQ-Swe, PRWE and VAS. N = 78

		VAS	PRWE		
			Pain subscale	Activity subscale	Total
**MHQ-Swe subscales**	I. Overall hand function	− .425**	− .496**	− .546**	− .555**
	II. ADL	− .433**	− .551**	− .786**	− .744**
	III. Work performance	− .402**	− .453**	− .557**	− .565**
	IV. Pain	− .674**	− .737**	− .550**	− .667**
	V. Aesthetics	− .312**	− .417**	− .402**	− .437**
	VI. Satisfaction	− .500**	− .568**	− .641**	− .676**
	Total score	− .553**	− .658**	− .719**	− .754**

**Correlation is significant at the 0.01 level (2-tailed)

## Discussion

The purpose of the study was to translate and culturally adapt the MHQ to Swedish and to test the validity and reliability in Swedish patients with surgically treated distal radius fractures. No major issues were revealed in the process of translation and cultural adaptation and the MHQ-Swe showed good validity and reliability in patients with surgically treated distal radius fractures.

The internal consistency of the subscales was high (0.77–0.96) and, despite being slightly lower than the original MHQ, it is still reflected (0.86–0.97) [9]. The high internal consistency indicates that the questions on the same subscale are correlated to one another and measure the same construct.

Test-retest reliability was tested with the ICC. The ICC was excellent on all subscales, slightly lower, but still comparable to the original questionnaire (0.77–0.90 in the MHQ-Swe compared with 0.81–0.97 in the original questionnaire) [9]. To assess the validity of the MHQ-Swe, we used the Swedish version of the PRWE, a commonly used questionnaire for this patient group, both in Sweden and in many other countries. Most other translation studies have used the Disabilities of the arm, shoulder and hand (DASH) for comparisons [13, 15, 27]. The PRWE was developed for outcome measurements after distal radius fractures, which made it a natural choice, even if the choice of using the PRWE makes the comparison with other translations more difficult. All the subscales in the MHQ-Swe showed moderate to strong correlations to the PRWE subscales, with the strongest correlation in the ADL and pain subscales, in accordance with Hulkkonen et al. [27] in the Finnish translation of the MHQ, although the DASH was used in the Finnish study. The high correlations in the subscales can be due to similar questions in the subscales. The lower correlations between the aesthetics subscale and the PRWE subscales were to be expected, since the appearance aspect is lacking in the PRWE. The presence of the aesthetic subscale is an advantage when assessing patients with hand deformities due to injuries or disease, for example, but it might not be as applicable in the distal radius fracture patient group.

A distal radius fracture is a temporary and hopefully transient injury, but it can be expected to affect a person’s involvement in daily activities for a limited time, and can therefore be called an occupational disruption. An occupational disruption is described by Whiteford [28] as “a temporary state, characterised by a significant disruption of identity associated with changes in the quantity and/or quality of one’s occupations subsequent to a significant life event, transition, or illness or injury. It has the potential to affect multiple areas of functioning, including social and emotional functioning” [28]. Together with the patient, the occupational therapist is able to identify the affected activities, facilitate participation in activities and ensure that the environment facilitates everyday life for the patient and counteracts negative consequences in life. Moreover, in the process of setting goals and performing and evaluating interventions, the MHQ-Swe could be a valuable tool. In terms of the items on the subscales, the questionnaire is holistic and covers most parts of human life, even though the questionnaire lacks components of leisure, which is an important part of our daily life. This can also be a limitation for some patients, who use leisure activities in goal setting.

The hands and their function are complex and very important for human beings, which makes a multifaceted evaluation questionnaire important. In this way, the MHQ has several favourable components. It is valuable to employ a questionnaire that has a holistic approach towards assessing variables related to hand injuries. The questionnaire includes aesthetics, something that is lacking in most questionnaires, and this could be important for many patients. The questionnaire also takes patient satisfaction with different aspects on the hand and its function into account, which is highly relevant. The questionnaire takes both hands into account, which is valuable, first and foremost for bilateral injuries and diseases, but this could also be somewhat confusing for the patient group in the study. Several patients did not fill in the function of the non-injured hand, which resulted in missing data, but, as we chose only to analyse the injured hand, this was not regarded as a major issue.

The MHQ scoring algorithm is somewhat complicated, as a few of the questions on the subscales, as well as an entire subscale (IV), have to be reversed to obtain a total score using the questionnaire. The reversed questions also confused some of the patients. For example, in terms of the aesthetic subscale (V), some patients did not read the questions in full and answered the opposite of what they should have answered. The first question on the subscale is the reverse of the other three questions and this may have resulted in some of the participants misunderstanding it and giving contradictory answers. One suggestion for improving the user friendliness would be to create an electronical version, with an automatic scoring algorithm, perhaps one for unilateral injuries and one for bilateral.

In terms of phase 1, where a group of patients completed the form and then answered questions about the questionnaire, it is possible to discuss whether the answers and feedback would have been different if we had conducted interviews instead of giving the respondents a form to fill in. This could be a limitation to the design and cultural adaptation of the questionnaire. In retrospect, we could also have added a question about the patient’s perception of the time consumption of the questionnaire and a question about its relevance to the current injury. The fact that we only calculated the results for one hand in the study is also a limitation.

## Conclusion

The MHQ-Swe is an appropriate and relevant PRO questionnaire, with good validity and reliability, for use in patients with surgically treated distal radius fractures.

## Data Availability

The datasets generated during and/or analysed during the current study are available from the corresponding author on reasonable request.

## Abbreviations

ADL: Activities of daily living
DRF: Distal radius fracture
MHQ: Michigan Hand Outcomes Questionnaire
ICC: Intraclass correlation coefficient
PROM: Patient-reported outcome measurements
PRWE: Patient rated wrist evaluation
ROM: Range of motion
VAS: Visual analogue scale
